# Pattern Separation: A Potential Marker of Impaired Hippocampal Adult Neurogenesis in Major Depressive Disorder

**DOI:** 10.3389/fnins.2017.00571

**Published:** 2017-10-26

**Authors:** Kellen Gandy, Sohye Kim, Carla Sharp, Lilian Dindo, Mirjana Maletic-Savatic, Chadi Calarge

**Affiliations:** ^1^Menninger Department of Psychiatry and Behavioral Sciences, Baylor College of Medicine, Houston, TX, United States; ^2^Department of Obstetrics and Gynecology, Baylor College of Medicine and Center for Reproductive Psychiatry, Pavilion for Women, Texas Children's Hospital, Houston, TX, United States; ^3^Department of Pediatrics, Baylor College of Medicine, Texas Children's Hospital, Houston, TX, United States; ^4^Department of Psychology, University of Houston, Houston, TX, United States; ^5^Dan and Jan Duncan Neurological Research Institute at Texas Children's Hospital, Houston, TX, United States

**Keywords:** neurogenesis, pattern separation, major depressive disorder, dentate gyrus, RDoC matrix, psychological inflexibility, emotional dysfunction

## Abstract

Adult neurogenesis involves the generation of new neurons, particularly in the dentate gyrus of the hippocampus. Decreased hippocampal neurogenesis has been implicated in both animal models of depression and in patients with major depressive disorder (MDD), despite some inconsistency in the literature. Here, we build upon current models to generate a new testable hypothesis, linking impaired neurogenesis to downstream psychological outcomes commonly observed in MDD. We contend that disruption in adult neurogenesis impairs pattern separation, a hippocampus-dependent function requiring the careful discrimination and storage of highly similar, but not identical, sensory inputs. This, in turn, can affect downstream processing and response selection, of relevance to emotional wellbeing. Specifically, disrupted pattern separation leads to misperceived stimuli (i.e., stimulus confusion), triggering the selection and deployment of established responses inappropriate for the actual stimuli. We speculate that this may be akin to activation of automatic thoughts, described in the Cognitive Behavior Theory of MDD. Similarly, this impaired ability to discriminate information at a fundamental sensory processing level (e.g., impaired pattern separation) could underlie impaired psychological flexibility, a core component of Acceptance and Commitment Therapy of MDD. We propose that research is needed to test this model by examining the relationship between cognitive functioning (e.g., pattern separation ability), psychological processes (e.g., perseveration and psychological inflexibility), and neurogenesis, taking advantage of emerging magnetic resonance spectroscopy-based imaging that measures neurogenesis *in-vivo*.

## Introduction

Major depressive disorder (MDD) is characterized by a triad of mood, neuro-vegetative, and cognitive symptoms (American Psychiatric Association, 2013). MDD is most prevalent among young adults (18–25 years old), particularly women, and is the second leading cause of disability worldwide (National Survey on Drug Use Health, 2015). Despite significant progress over the last few decades, treatment efficacy remains sub-optimal for the majority of patients with MDD (National Institute of Mental Health, 2015). Moreover, research has yet to fully elucidate the underlying pathophysiology. This may not be surprising, given the complexity and clinical heterogeneity of MDD.

In this paper, we build upon current models (Hanson et al., 2011; Shelton and Kirwan, 2013; Hill et al., 2015; Lucassen et al., 2015; Miller and Hen, 2015; Yun et al., 2016) to propose that reduced adult neurogenesis in the dentate gyrus of the hippocampus causes deficits in pattern separation and downstream impairment in information processing. This, in turn, contributes to MDD presentation, at least in a subgroup of patients. We begin by reviewing the nascent literature on hippocampal neurogenesis in MDD. We then link neurogenesis and pattern separation in the context of MDD, and complete the review with a speculation regarding the potential downstream impairment in information processing contributing to cognitive patterns characteristic of patients with MDD.

## Hippocampal neurogenesis and MDD

Neurogenesis involves generating new, functional neurons. As such, it has traditionally been thought to occur only during embryogenesis and the perinatal stages of the mammalian nervous system development. However, over the past two decades, research has firmly established that newborn neurons are generated in two germinal zones of the postnatal and adult brain of rodents as well as primates, including humans: the subgranular zone of the dentate gyrus of the hippocampus (Altman, 1962; Palmer et al., 1997; Eriksson et al., 1998; Knoth et al., 2010; Miller et al., 2013) and the subventricular zone of the lateral ventricles (Morshead et al., 1994; Doetsch et al., 1997; Quinones-Hinojosa et al., 2006; Bergmann et al., 2012; Curtis et al., 2012). Adult-generated neurons form synaptic connections and integrate into the local circuitry. In the dentate gyrus, it is estimated that about 9,000 newborn neurons are generated daily in the adult rat, replacing about 40% of the structure over the life-span (Snyder and Cameron, 2012). In humans, carbon-dating estimated that about 700 newborn neurons are added to the hippocampal adult circuitry daily, replacing about 30% of the structure over the life-span (Spalding et al., 2013). These data indicate that the number of new neurons incorporated into the hippocampal circuitry in the adult brain is likely to be large enough to affect hippocampal function both in rodents and in humans. Importantly, based on animal studies, these new neurons participate in the modulation and refinement of established neuronal circuitry, affecting both regional physiology and the functional connectivity of more distant brain regions, such as the prefrontal cortex, amygdala, and other structures within the limbic system (van Praag et al., 2002; Ramirez-Amaya et al., 2006; Toni et al., 2007, 2008; Vivar et al., 2012; Vivar and van Praag, 2013). The integration of these new neurons into the hippocampal circuitry suggests an important role for adult neurogenesis in hippocampus-dependent functions. For instance, newly-generated neurons in the murine dentate gyrus contribute to the encoding of new memories (Farioli-Vecchioli et al., 2008; Jessberger et al., 2009), spatial learning (Snyder et al., 2005; Dupret et al., 2008; Clelland et al., 2009), pattern separation (Sahay et al., 2011a,b), affect regulation (Ibi et al., 2008), and cognitive flexibility (Burghardt et al., 2012), which, coincidentally, can all be affected in individuals diagnosed with MDD (Bremner et al., 2004; Deveney and Deldin, 2006; Gould et al., 2007; Joormann and Gotlib, 2010; Shelton and Kirwan, 2013).

Dysregulated neurogenesis may contribute to MDD, anxiety and other neuropsychiatric disorders (Lucassen et al., 2015). Due to the lack of precise animal models of MDD, studies utilize different stressors to induce depressive-like states. In rodents, both acute psychosocial stress (e.g., exposure to a social dominance paradigm, social instability or social isolation), as well as chronic stress reduce hippocampal neurogenesis (Thomas et al., 2007; Brummelte and Galea, 2010; Castilla-Ortega et al., 2011; McCormick et al., 2012). Similarly, social isolation-stress in primates decreases hippocampal neurogenesis and concurrently induces depressive and anxiety-like phenotypes, including anhedonia and self-defeating behavior (Perera et al., 2011). Moreover, the lasting effects of chronic stress during early life include the inhibition of adult neurogenesis (Karten et al., 2005; Korosi et al., 2012), and potentiation of anxiety-like behaviors (de Andrade et al., 2013). However, stress-related effects are dose-dependent, and a “short” exposure to “weaker” stressors may not affect hippocampal neurogenesis (Kempermann, 2002).

While the debate on the association of MDD and hippocampal adult neurogenesis continues (Boldrini et al., 2009, 2013; Hayes et al., 2013; Huang et al., 2013; Wu et al., 2014; Miller and Hen, 2015), the most convincing data on this association comes from studies examining the impact of interventions with anti-depressant potential on neurogenesis. In fact, therapeutic interventions that promote mental well-being stimulate hippocampal neurogenesis. For instance, routine aerobic exercise reduces learned helplessness and depressive-like behaviors (i.e., sucrose preference, forced swim test, etc.) in animals (Binder et al., 2004; Yau et al., 2011; Liu et al., 2013), endogenous corticosterone (Starzec et al., 1983), stress-mediated responses from the hypothalamic-pituitary-adrenal (HPA) axis (Luger et al., 1987; Campeau et al., 2010), and also promotes neurogenesis in the dentate gyrus (van Praag et al., 1999; Bjornebekk et al., 2005; Kronenberg et al., 2006; Marlatt et al., 2012; Dery et al., 2013). Similarly, environmental enrichment enhances the proliferation of neural stem cells in the dentate gyrus of mice, while concurrently improving depressive-like behaviors (Kempermann, 2002; Veena et al., 2009a,b; Jha et al., 2011). Finally, antidepressant treatments, such as SSRIs and electroconvulsive shock (equivalent to human electroconvulsive therapy, ECT) increase neurogenesis specifically in the hippocampus and not in other neurogenic regions (Santarelli et al., 2003; Kodama et al., 2004; David et al., 2009; Klomp et al., 2014). In fact, animal research indicates that electroconvulsive shock is one of the strongest stimuli for hippocampal adult neurogenesis (Madsen et al., 2005; Warner-Schmidt et al., 2008; Chen et al., 2009). In rats, even a single electroconvulsive shock can increase neurogenesis by 67–197% (Segi-Nishida et al., 2008; Chen et al., 2009). In clinical practice, ECT is administered as a series of treatments over a number of weeks. The closest analog to this paradigm examined the effect of electroconvulsive stimulations in adult monkeys and found a 4-fold increase in dentate subgranular zone cell proliferation (Santarelli et al., 2003; Perera et al., 2007). Notably, the maturation period of newly-generated neurons in the dentate gyrus appears consistent with the delay for the full therapeutic effects of antidepressants to become manifest (Esposito et al., 2005; Ngwenya et al., 2006). In sum, these preclinical findings suggest that adult neurogenesis may be modulated by factors associated with MDD, including chronic stress (Cohen et al., 2007), and activation of the HPA axis (Pariante and Lightman, 2008).

While pre-clinical findings do not always translate to humans, research has also found evidence potentially implicating impaired hippocampal neurogenesis in MDD. First, MDD has long been associated with abnormalities in the limbic system, including the hippocampus (MacQueen et al., 2003; Whittle et al., 2014). This has been highlighted in a recent meta-analysis reporting that smaller hippocampal volumes are the most consistent sub-cortical abnormality in patients with MDD, particularly adolescents and emerging adults (i.e., <21 years old), as well as in those with recurrent MDD (Schmaal et al., 2016). More specifically, high-resolution volumetric magnetic resonance imaging (MRI) and postmortem studies have found decreased dentate gyrus size in unmedicated patients with MDD (Boldrini et al., 2009, 2013; Huang et al., 2013). In fact, the number of granule cells, derived from neural progenitor cells, was smaller in the anterior and mid regions of the dentate gyrus of untreated MDD patients (Boldrini et al., 2013). Interestingly, for untreated MDD, younger age of MDD onset correlated with fewer granule cells in the anterior dentate gyrus. Moreover, untreated patients with MDD appear to have a smaller number of dividing cells in the dentate gyrus compared to healthy controls (Boldrini et al., 2009). Of note, while this finding was not significant likely due to lack of statistical power (*n* = 5 for unmedicated MDD, *n* = 7 for healthy controls), the group differences were quite large (Cohen's *d* effect size ≥ 1.2). Although the exact mechanism is not yet known, these findings suggest that cell division and granule cell survival in the dentate gyrus are reduced in unmedicated MDD patients. Nonetheless, the number of cells generated by adult neurogenesis and their corresponding volume cannot entirely account for the observed change in hippocampal volume in patients with MDD. In fact, hippocampal volume in patients with MDD is likely the result of various factors, including reduced neuronal number and size, synaptic density, dendritic complexity, axonal hypotrophy and glial cell density (Stockmeier et al., 2004; Duman et al., 2016). Rather, associated changes in local brain circuitry and glial cells, including secondary apoptosis, could follow impaired neurogenesis in MDD, resulting in the volumetric differences (Wiskott et al., 2006; Kubera et al., 2011; Lee et al., 2012). Of course, additional research is needed to more convincingly determine whether and to which extent neurogenesis is impaired in MDD and how this contributes to the observed volumetric and functional brain changes.

While the evidence reviewed above suggests the presence of a link between reduced hippocampal adult neurogenesis and MDD, preclinical and clinical studies have also reported findings that are inconsistent with this hypothesis (Miller and Hen, 2015). For instance, exposure to several stress models failed to reduce hippocampal neurogenesis in rodents (Hanson et al., 2011). Moreover, depressive-like symptoms in rodents can improve without change in hippocampal neurogenesis (Meshi et al., 2006; Bessa et al., 2009). Similarly, depressive-like behaviors in rodents improve following antidepressant treatment, despite ablated hippocampal neurogenesis (Cowen et al., 2008; Holick et al., 2008; Huang et al., 2008; David et al., 2009). Finally, not all postmortem studies found reduced neurogenesis in patients with MDD (Reif et al., 2006). However, these seemingly contradictory findings may rather reflect differences in the genetic strains of the rodents studied (Semerci and Maletic-Savatic, 2016), the paradigms used to induce depressive-like behaviors in the lab (e.g., unpredictable mild stress, cortisol-induced depression), or the behaviors used as markers of depressive-like states in animals (e.g., sucrose preference test, learned helplessness, and forced swim test) (Santarelli et al., 2003; Bjornebekk et al., 2005; Meshi et al., 2006; Bessa et al., 2009; Yau et al., 2014). In human studies, mixed results could also reflect methodological differences given that different biomarkers of adult neurogenesis exist, with varying sensitivity (Reif et al., 2006; Boldrini et al., 2009). Additionally, the presence of inconsistent findings could also reflect the fact that neurogenesis may be sufficient but not necessary for the development of depression or for antidepressants to be efficacious. Moreover, decreased neurogenesis may be associated with only certain characteristics of MDD or with a subgroup of patients with MDD, given the multifactorial nature of this disorder. For example, while in preclinical research, hippocampal adult neurogenesis could be virtually completely aborted experimentally, it can be affected to varying degrees in patients with MDD, depending on etiology, severity, subtype, and comorbidity. Finally, it is also important to keep in mind that evolutionary pressures may have led to very different roles played by hippocampal adult neurogenesis in the human brain compared to that of a rodent.

## Pattern separation as a cognitive marker of adult neurogenesis

The ability to discriminate and store similar, but not identical, inputs of sensory information into distinct representations (e.g., form distinct memories) is referred to as “pattern separation.” This function is notable for its dependence on hippocampal adult neurogenesis (Aimone et al., 2011). In fact, rodents with ablated neurogenesis in the dentate gyrus display impairments in pattern separation ability (Clelland et al., 2009). In contrast, increasing hippocampal neurogenesis leads to enhanced pattern separation ability in animals (Sahay et al., 2011a). Hippocampal neurogenesis is also implicated in a variety of additional processes, including cognitive flexibility (Burghardt et al., 2012), hippocampus-dependent memory functions (Winocur et al., 2006), spatial memory (Snyder et al., 2005; Dupret et al., 2008; Clelland et al., 2009), memory encoding (Epp et al., 2016), and executive function (Saxe et al., 2007). However, whether its role is required for these functions remains to be determined (Cushman et al., 2012; Groves et al., 2013; Swan et al., 2014; Park et al., 2015; Svensson et al., 2016). A significant challenge in this research is determining the magnitude of pattern separation demanded by each of these cognitive task. It is those tasks that manipulate the level of sensory discrimination by altering the degree of similarity among study items that appear to most strongly correlate with neurogenesis in the dentate gyrus (Hvoslef-Eide and Oomen, 2016).

In humans, experimental tasks which place a high demand on sensory discrimination have been correlated with dentate gyrus activity in healthy controls. Kirwan and Stark (2007) developed a mnemonic similarity task that involves discriminating the visual similarities of two different, but similar, images. Increased performance on pattern separation while completing this task was associated with increased blood oxygen level-dependent (BOLD) signal in the dentate gyrus and CA3 region of the hippocampus (Kirwan and Stark, 2007; Yassa and Stark, 2011). Additionally, changes in dentate gyrus activity correlate with the degree of mnemonic discrimination, with highly similar lures resulting in increased BOLD signaling (Bakker et al., 2008; Lacy et al., 2011). Moreover, Déry et al. found that aerobic exercise, which is known to promote adult neurogenesis, was prospectively associated with improved performance on the mnemonic similarity task (Dery et al., 2013). In addition, they observed a concurrent decline in depressive symptoms (Dery et al., 2013). This is consistent with findings from a study in college students, whereby performance on the same task was inversely correlated with depression severity, as captured by the Beck Depression Inventory (Shelton and Kirwan, 2013). This should not be surprising in light of evidence showing poor performance on hippocampus-dependent tasks in MDD (MacQueen et al., 2003).

Additionally, MDD patients consistently display impairments in long-term memory (Burt et al., 1995; Soderlund et al., 2014), working memory (Rose and Ebmeier, 2006), negative emotional bias (Gotlib and Joormann, 2010) and executive function, including problem solving, attentional control, planning, and cognitive inhibition (Frodl et al., 2006; Letkiewicz et al., 2014). These deficits in executive functioning are positively associated with depression severity (Snyder, 2013) and are typically accompanied by structural and functional brain abnormalities in the prefrontal cortex, ventromedial basal ganglia, amygdala, and hippocampus (Frodl et al., 2006; Drevets et al., 2008). Thus, while to our knowledge tasks that specifically activate the dentate gyrus have not been directly examined in MDD, the available evidence suggests performance would be suboptimal. To what extent such impairment is specific to MDD would require further investigation given that disrupted pattern separation ability has been observed in schizophrenia (Das et al., 2014), mild cognitive impairment (Stark et al., 2013), and amnesia (Kirwan et al., 2012). Of note, these studies relied exclusively on behavioral data without a neuroimaging component, making it difficult to establish in humans the direct involvement of the hippocampus in general, or the dentate gyrus in particular, in pattern separation.

## Deficit in information processing as a sequelae of pattern separation impairment in MDD

As previously noted, adult neurogenesis in the dentate gyrus is necessary for the discrimination of new sensory information (e.g., pattern separation). Thus, impaired pattern separation may hamper one's ability to process new information. We speculate that this may explain some of the phenomena observed in patients with MDD. For instance, individuals with impaired pattern separation ability could mistake comparable stimuli as being identical which, in turn, may lead to these distinct stimuli triggering the same response (e.g., responding with sadness to both negative and ambiguous events). In fact, individuals diagnosed with or at-risk for depression and anxiety disorders tend to interpret ambiguous stimuli as threatening or negative, further supporting the hypothesis that pattern separation may be deficient in MDD (Leppanen et al., 2004; Mogg et al., 2006; Dearing and Gotlib, 2009). We contend that an impaired ability to discriminate information at the fundamental sensory processing level, in conjunction with a tendency to over-generalize information, could underlie ruminative thinking, perseverative or inflexible behavior, and cognitive rigidity; all of which are common in MDD (Watkins and Teasdale, 2001; Marazziti et al., 2010). As such, it could explain the mechanism underlying the activation of “automatic thoughts” or “schemas,” described in the Cognitive Behavioral Therapy model of MDD (Beck, 1979). For instance, the inability to identify discrepancies between stimuli may lead to stimulus “confusion,” triggering “responses” rehearsed and reinforced in overlapping but not identical situations. When these reflexive “responses” are cognitive, they are akin to automatic thoughts. Such stereotypic responses to situations could also disrupt psychological flexibility, highlighted in Acceptance and Commitment Therapy as a core process (Hayes et al., 2013). It refers to one's propensity to willingly select behavioral responses based on his/her chosen values, rather than reflexively reverting to familiar actions (e.g., maladaptive habits), that may provide short-term relief without regard to the long-term ramifications. In fact, inflexibility related to impaired pattern separation may also extend to social interactions and relationships where the inability to take the perspective of others and adequately reflect on one's own motives, thoughts, desires and feelings are described as mentalizing deficits (Fonagy, 2003; Fischer-Kern et al., 2013). In this sense, neurogenesis, via pattern separation, may be critical for the development of metacognitive function, with clear implications for psychological well-being.

Finally, hippocampal neurogenesis also appears to contribute to emotional regulation (Femenia et al., 2012). The psychological regulation of emotions is a complex process, dependent on widespread neural networks, involving the limbic system, prefrontal cortex, amygdala and hippocampus (Davidson and Irwin, 1999; Lane et al., 2000; Phan et al., 2002). The ability to regulate one's emotions has been repeatedly found to be impaired in MDD (Gotlib and Joormann, 2010). This, again, highlights the potential functional impact of hippocampal adult neurogenesis in modulating local and more distant brain circuitry, including that involved in emotion regulation.

In sum, we speculate that impaired neurogenesis in MDD disrupts performance in pattern separation. This, in turn, affects higher-level processes resulting in cognitive and behavioral rigidity thought to manifest in ruminative thinking, activation of automatic thoughts and schemas, psychological inflexibility, and deficient mentalizing. Future studies in MDD should, therefore, aim not only to examine the association between adult neurogenesis and behavioral performance on pattern separation tasks, but also strive to investigate its association with the functioning of higher-order psychological processes implicated in MDD (e.g., psychological inflexibility, ruminative thinking, and mentalizing). As such, a multi-level assessment could be undertaken with different units of analysis (Table 1), similar to what has been proposed in the Research Domain Criteria matrix (Cuthbert and Insel, 2013).

**Table 1:** Proposed units of analysis to examine the role of pattern separation in depressive and anxiety disorders, presented in a Research Domain Criteria (RDoC) matrix format (Cuthbert and Insel, 2013).

**Construct**	**Molecules**	**Cells**	**Circuits**	**Behavior**	**Self-Report**
Pattern Separation	Mono-Unsaturated Fatty Acids Resonating at 1.28 ppm	Neurogenesis	Frontal–Hippocampal–Dentate Gyrus–Limbic System	Mnemonic Similarity Task	^*^BDI, AAQ, YSQ, RFQ

The RDoC was introduced by the National Institute of Mental Health as a novel research framework to study psychopathology. It integrates several units of analysis spanning from the basic genetic/molecular level to the behavioral level.

* *BDI, Beck Depression Inventory; AAQ, Acceptance and Action Questionnaire; YSQ, Young Schema Questionnaire; RFQ, Reflective Functioning Questionnaire*.

## Conclusion

This review proposes that reduced adult neurogenesis in the dentate gyrus causes deficits in pattern separation and downstream impairment in intra- and interpersonal information processing, thus forming one of the mechanisms underlying MDD and perhaps antidepressant efficacy. As such, future studies should build on available findings from non-clinical samples linking performance on pattern separation tasks to depression severity in order to determine its association with psychological functioning implicated in depressive and anxiety disorders, such as catastrophizing, impaired psychological flexibility, and mentalizing deficit (Beck et al., 1961; Young, 1994; Fonagy, 2003; Hayes et al., 2013). This could be combined with emerging state-of-the-art technology to assess neurogenesis *in-vivo* in humans. In fact, magnetic resonance spectroscopy (MRS)-based imaging is making progress toward this goal, measuring mono-unsaturated fatty acids highly enriched in neuroprogenitor cells that resonate at 1.28 ppm in the NMR spectrum (Ma et al., 2011; Choi et al., 2017). While providing only an indirect measure of neurogenesis, this state-of-the-art MRS-based technique will be a valuable tool to supplement other advances recently made in *in-vivo* imaging of hippocampal adult neurogenesis in humans (Sierra et al., 2011; Ho et al., 2013; Tamura and Kataoka, 2017; Van de Bittner et al., 2017). Ultimately, any measure of hippocampal neurogenesis would need to be combined with measures of functional brain activity in order to provide further validation of the model we propose.

## Author contributions

KG, MM, and CC conceptualized this work and prepared the first draft. SK, CS, and LD provided revisions for important intellectual content. All authors gave final approval of the manuscript to be published and have agreed to be held accountable for the accuracy and integrity of this manuscript.

### Conflict of interest statement

The authors declare that the research was conducted in the absence of any commercial or financial relationships that could be construed as a potential conflict of interest.
